# Occupational Traumatic Amputation of Distal Part of Thumb Caused by Constriction Effect of Nylon Rope: A Case Report with Review of Literature

**DOI:** 10.1155/2011/427823

**Published:** 2011-10-09

**Authors:** M. Kalra, A. Mahmood, M. Patralekh

**Affiliations:** ^1^Department of Orthopaedics, Lady Hardinge Medical College, Delhi 110001, India; ^2^Department of Orthopaedics, Sohar Hospital, Sohar 321, Oman; ^3^Central Health Services, C1-160/161, 2nd Floor, Sector16, Rohini, Delhi 110089, India

## Abstract

A 60-year-old farmer came to the accident and emergency department complaining of traumatic amputation of distal part of right thumb. On examination, there was a circumferential wound located at the base of right thumbnail with injury to both neurovascular bundles and fracture of distal part of distal phalanx with the distal part hanging by volar skin only. The wound was irrigated and the distal part terminalised with direct loose skin closure. Discussion with the patient about the scenario of trauma revealed a mechanism of injury which may appear unique.

## 1. Introduction

Extremity traumatic amputation, particularly of thumb, is a potentially devastating event in a person's life and often resulting in profound physical, psychological, and vocational consequences. Trauma related amputation is the second most common cause of extremity loss and occurs mostly in productive age of less than fifty. Traumatic amputation usually results directly from work-related (occupational) injury and from factory (industrial), farm (agricultural), or power tool accidents. It may also be caused from nonwork related (nonoccupational) injury, from motor-vehicle accidents, housework, and crush injuries. Natural disaster, war, and terrorist attacks can also cause traumatic amputation [1–3]. We are presenting here a case of distal thumb amputation in a farmer with an interesting mechanism of injury.

## 2. Case Report

**Figure 1:**
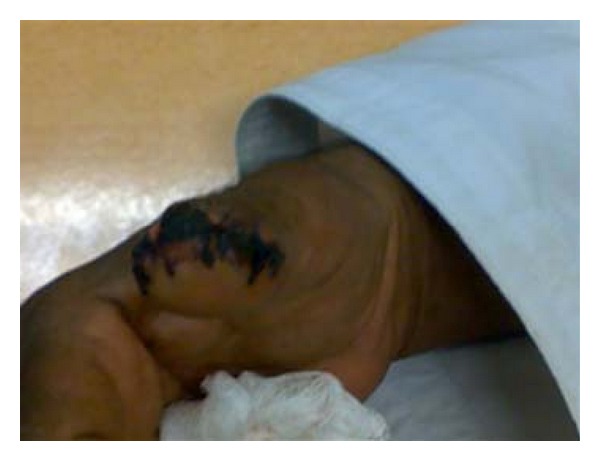
Amputated distal thumb and loose closure done.

A 60-year-old farmer came to the accident and emergency department complaining of traumatic amputation of distal part of right thumb. On examination, there was a circumferential wound located at the base of right thumbnail with injury to both neurovascular bundles and fracture of distal part of distal phalanx with the distal part hanging by volar skin only. The wound was irrigated and the distal part terminalised with direct loose skin closure (Figure 1). Patient was admitted for wound dressings and IV antibiotics for 48 hours after which he was discharged. The patient was seen in OPD after 1 week and was found to suffer from superficial wound infection that settled with oral antibiotics. He was followed up after 3 days (10 days from the day of trauma) when the stitches were removed. The wound was completely healed.

**Figure 2:**
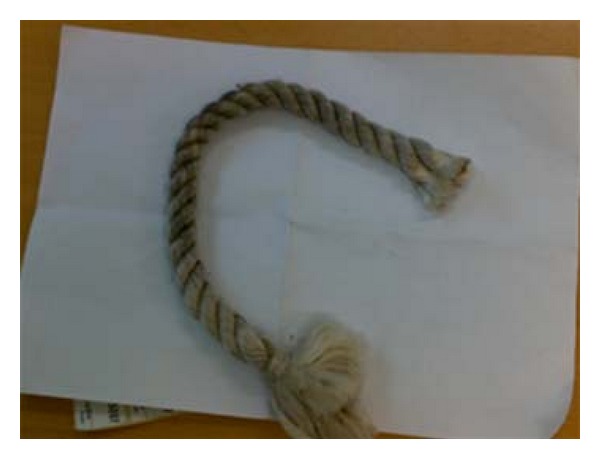
The nylon rope.

**Figure 3:**
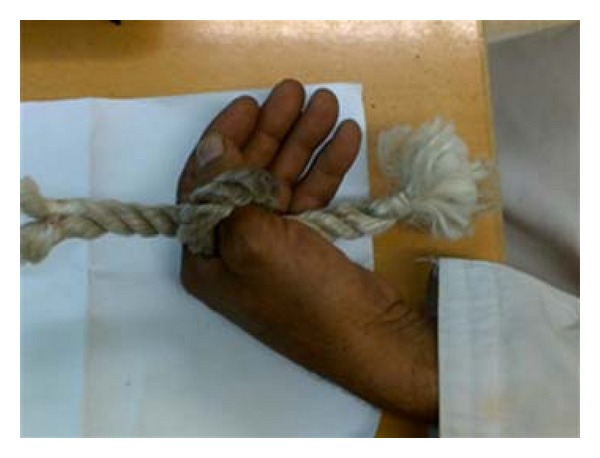
Demonstration of mechanism of injury.

On discussion with the patient about the scenario of trauma, it was found that as a farmer, in order to slaughter an animal, he used to tie a nylon rope around the neck of the animal and anchor the other end around his thumb so as to stabilize the animal in order to proceed with the slaughter. In the present instance, the animal while trying to escape, stretched the other end of the rope causing severe constriction of the farmers thumb resulting in its near total amputation. Probably, the nylon material acted as a knife cutting the farmers thumb. Nylon rope and probable mechanism of injury is demonstrated in Figures 2 and 3. Informed consent was taken from the patient that his case will be considered for publication in an international journal including electronic publication on the internet.

## 3. Discussion

*Functions of thumb*. Thumb is probably the most important part of human hand. From evolutionary perspective, development of thumb opposability is considered to be as important as that of human brain. The thumb is important in the formation of a grip.

One of the earlier significant contributors to the study of hand grips was anatomist and anthropologist John Russell Napier who proposed organizing the movements of the hand by their anatomical and functional basis, as opposed to work done earlier that had only used arbitrary classification. Most of this early work on hand grips had a pragmatic basis as it was intended to narrowly define compensable injuries to the hand, which required an understanding of the anatomical basis of hand movement. Napier proposed two primary prehensile grips: the *precision grip *and the *power grip*. The precision and power grip are defined by the position of the thumb and fingers, where we find the following:

- The power grip is when the fingers (and sometimes palm) clamp down on an object with the thumb makes counter pressure. Examples of the power grip are gripping a hammer, opening a jar using *both your palm and fingers*, and during pull-ups.
- The precision grip is when the intermediate and distal phalanges (“fingertips”) and the thumb press against each other. Examples of a precision grip are writing with a pencil, opening a jar *with the fingertips alone*, and gripping a ball (as long as it is not tight against your palm) [4]. Understandably, thumb plays an important role in both these types of grips. Thumb amputations have been given code occupational diseases code 031(0311,0319) in Occupational Injury and Illness Classification Manual (U.S. Department of Labor Bureau of Labor Statistics), 885.0 (without complication), and 885.1 (with complication) as per WHO ICD9, thereby giving it special importance and status [5, 6].

**Management**

Thumb amputations can severely affect hand function. The anatomical level of amputation determines the extent of the functional deficit, so preserving thumb length is a more critical factor than mobility. The attributes that make the thumb unique are position, stability, strength, length, motion, sensibility, and appearance. Of these qualities, the first four must be present to an acceptable extent for function to approach normality, while the latter three are very desirable but not essential. Reconstructive alternatives following amputation can be considered in four broad groups: (a) where the length is acceptable but the covering is poor, (b) subtotal amputation, where length is equivocal, (c) total amputation with the basal joint preserved, and (d) total amputation with the basal joint destroyed. In the first group, soft-tissue cover can be improved by local flaps (as in our case) with or without a neurovascular pedicle or by microvascular free pulp transfer. In the second group, metacarpal lengthening by distraction, with or without phalangization, may give adequate length. In total amputations, one may choose osteoplastic reconstruction, pollicisation, or toe-to-hand transfer. Which solution is selected depends on the level of the amputation, the presence and nature of injuries to other digits, occupational and social factors, and the availability of tissues. Replantation usually provides the best return to function but is not always possible due to extent of injury to the severed digit or the hand.

**Epidemiology**

Traumatic amputation is seen with increasing frequency from the second to fifth decades of life. Liang et al. [7] reported young male manufacturing workers were at high risk of occupational amputation of upper extremities. But Dillingham et al. [2] reported increasing risk of amputation on those older than 85 years. Conn et al. [8] observed greatest risk of nonwork related finger amputation in young children and older adults in the United States.

More than 60% of cases sustained occupation-related injury with high frequency in industrial and agricultural works [9]. This finding is compatible with the results of Boyle et al. [10], Onuba [11] and Stanbury et al. [12] researches. In the nonoccupational group motor vehicle accident was the most common cause (24.5%) [1, 8]. With regard to temporal distribution, traumatic amputation was more frequent in the spring and autumn and in the May and September. Spring and autumn are planting and harvesting time, and May and September are the peak of planting and harvesting activities, respectively, in our region. It was reported that there are common patterns of traumatic amputations in children based on the mechanism of injury, the season, and the age of the child [13]. A study from Hansen [14] showed highest incidence of major injury due to agricultural machinery during spring planting (May through June) and fall harvesting (September through October) time.

Hansen and Carstensen [15] demonstrated that in agricultural machinery injury, upper extremity was the most common site of injury. The hand and its distal part (finger) sustained the most common traumatic amputation. The same is true in our case though amputation was caused by constriction effect of nylon rope rather than machinery. Dillingham et al. [2] research resulted that half of all trauma related amputation occurred in the upper extremity and three quarter of all upper extremity traumatic amputation occurred in the lower part of upper extremity (finger). In the present study, terminal part of thumb was involved. Terzioglu et al. [16], showed hand injury of children with agricultural machinery was most commonly associated with injury of third digit, and the thumb was the least. Distal phalanx was amputated most commonly [8]. Doraiswamy and Baig [17] found terminal phalanx being the most common injured (not amputated) part in children.

It is concluded that extremity traumatic amputation was more prevalent in young male industrial and agricultural workers, motor vehicle accidents, spring and autumn seasons, and upper extremity especially its distal part. With regard to these epidemiological characteristics, accordingly, appropriate precaution measures may decline the incidence or decrease the severity of extremity traumatic amputation [9].

This is a case of occupational injury, which is a common occurrence in Oman caused by a faulty technique of animal slaughter by farmers along with wrong choice of material used for the rope. Probably it is chosen as it forms a firm grip around animal's neck, but also in the mean time, it proves to be very sharp on the farmers' thumb.

*It is our advice that in order to avoid such major disfiguring and incapacitating injury, there should be educational programs for farmers, which may be implemented by the ministry of agriculture, about safe techniques of animal slaughter, use of ropes made up of safer materials, and encouraging animal slaughter with modern methods not traditional ones.*
